# Supplementary material for: HIVprotI: an integrated web based platform for prediction and design of HIV proteins inhibitors
Source: J Cheminform. 2018 Mar 9;10:12. doi: 10.1186/s13321-018-0266-y (PMC5845081; doi:10.1186/s13321-018-0266-y)
Supplement: Supplementary file 2 — Additional file 2. Source code of HIVProtI web server. [file 13321_2018_266_MOESM2_ESM.zip › HIVprotI_Source-code/hivprotI/weka-3-6-6/doc/weka/attributeSelection/FilteredSubsetEval.html]

FilteredSubsetEval


---


|  |  |  |
| --- | --- | --- |
| **Overview** \| **Package** \| **Class** \| **Tree** \| **Deprecated** \| **Index** \| **Help** \| **Weka's home** | |  |
| **PREV CLASS**   **NEXT CLASS** | **FRAMES**    **NO FRAMES**     **All Classes** |  |
| SUMMARY: NESTED \| FIELD \| CONSTR \| METHOD | DETAIL: FIELD \| CONSTR \| METHOD |  |


---


## weka.attributeSelection Class FilteredSubsetEval

```
java.lang.Object
  weka.attributeSelection.ASEvaluation
      weka.attributeSelection.FilteredSubsetEval
```

**All Implemented Interfaces:**: java.io.Serializable, SubsetEvaluator, CapabilitiesHandler, OptionHandler, RevisionHandler

---

``` public class FilteredSubsetEval extends ASEvaluation implements java.io.Serializable, SubsetEvaluator, OptionHandler ```

Class for running an arbitrary subset evaluator on data that has been passed through an arbitrary
filter (note: filters that alter the order or number of attributes are not allowed).
Like the evaluator, the structure of the filter is based exclusively on the training data.


Valid options are:

```
 -W <evaluator specification>
  Full name of base evaluator to use, followed by evaluator options.
  eg: "weka.attributeSelection.CfsSubsetEval -L"
```

```
 -F <filter specification>
  Full class name of filter to use, followed
  by filter options.
  eg: "weka.filters.supervised.instance.SpreadSubsample -M 1"
```

**Version:**
:   $Revision: 5562 $

**Author:**
:   Mark Hall (mhall{[at]}pentaho{[dot]}com)

**See Also:**: Serialized Form

---

**Constructor Summary**

`FilteredSubsetEval()`


| **Method Summary** | |
| --- | --- |
| `void` | `buildEvaluator(Instances data)`             Initializes a filtered attribute evaluator. |
| `double` | `evaluateSubset(java.util.BitSet subset)`             evaluates a subset of attributes |
| `java.lang.String` | `filterTipText()`             Returns the tip text for this property |
| `Capabilities` | `getCapabilities()`             Returns default capabilities of the evaluator. |
| `Filter` | `getFilter()`             Get the filter to use |
| `java.lang.String[]` | `getOptions()`             Gets the current settings of the subset evaluator. |
| `java.lang.String` | `getRevision()`             Returns the revision string. |
| `ASEvaluation` | `getSubsetEvaluator()`             Get the subset evaluator to use |
| `java.lang.String` | `globalInfo()` |
| `java.util.Enumeration` | `listOptions()`             Returns an enumeration describing the available options. |
| `static void` | `main(java.lang.String[] args)`             Main method for testing this class. |
| `void` | `setFilter(Filter newFilter)`             Set the filter to use |
| `void` | `setOptions(java.lang.String[] options)`             Parses a given list of options. |
| `void` | `setSubsetEvaluator(ASEvaluation newEvaluator)`             Set the subset evaluator to use |
| `java.lang.String` | `subsetEvaluatorTipText()`             Returns the tip text for this property |
| `java.lang.String` | `toString()`             Describe the attribute evaluator |

**Methods inherited from class weka.attributeSelection.ASEvaluation**

`forName, makeCopies, postProcess`

**Methods inherited from class java.lang.Object**

`equals, getClass, hashCode, notify, notifyAll, wait, wait, wait`

**Constructor Detail**

### FilteredSubsetEval

```
public FilteredSubsetEval()
```


**Method Detail**

### getCapabilities

```
public Capabilities getCapabilities()
```

:   Returns default capabilities of the evaluator.

    :   **Specified by:**: `getCapabilities` in interface `CapabilitiesHandler` **Overrides:**: `getCapabilities` in class `ASEvaluation`
    :   **Returns:**: the capabilities of this evaluator. **See Also:**: `Capabilities`

---


### globalInfo

```
public java.lang.String globalInfo()
```

:   **Returns:**: a description of the evaluator suitable for displaying in the explorer/experimenter gui

---


### listOptions

```
public java.util.Enumeration listOptions()
```

:   Returns an enumeration describing the available options.

    :   **Specified by:**: `listOptions` in interface `OptionHandler`
    :   **Returns:**: an enumeration of all the available options.

---


### setOptions

```
public void setOptions(java.lang.String[] options)
                throws java.lang.Exception
```

:   Parses a given list of options.


    Valid options are:

    ```
     -W <evaluator specification>
      Full name of base evaluator to use, followed by evaluator options.
      eg: "weka.attributeSelection.CfsSubsetEval -L"
    ```

    ```
     -F <filter specification>
      Full class name of filter to use, followed
      by filter options.
      eg: "weka.filters.supervised.instance.SpreadSubsample -M 1"
    ```

    :   **Specified by:**: `setOptions` in interface `OptionHandler`
    :   **Parameters:**: `options` - the list of options as an array of strings **Throws:**: `java.lang.Exception` - if an option is not supported

---


### getOptions

```
public java.lang.String[] getOptions()
```

:   Gets the current settings of the subset evaluator.

    :   **Specified by:**: `getOptions` in interface `OptionHandler`
    :   **Returns:**: an array of strings suitable for passing to setOptions

---


### subsetEvaluatorTipText

```
public java.lang.String subsetEvaluatorTipText()
```

:   Returns the tip text for this property

    :   **Returns:**: tip text for this property suitable for displaying in the explorer/experimenter gui

---


### setSubsetEvaluator

```
public void setSubsetEvaluator(ASEvaluation newEvaluator)
```

:   Set the subset evaluator to use

    :   **Parameters:**: `newEvaluator` - the subset evaluator to use

---


### getSubsetEvaluator

```
public ASEvaluation getSubsetEvaluator()
```

:   Get the subset evaluator to use

    :   **Returns:**: the subset evaluator to use

---


### filterTipText

```
public java.lang.String filterTipText()
```

:   Returns the tip text for this property

    :   **Returns:**: tip text for this property suitable for displaying in the explorer/experimenter gui

---


### setFilter

```
public void setFilter(Filter newFilter)
```

:   Set the filter to use

    :   **Parameters:**: `newFilter` - the filter to use

---


### getFilter

```
public Filter getFilter()
```

:   Get the filter to use

    :   **Returns:**: the filter to use

---


### getRevision

```
public java.lang.String getRevision()
```

:   Returns the revision string.

    :   **Specified by:**: `getRevision` in interface `RevisionHandler` **Overrides:**: `getRevision` in class `ASEvaluation`
    :   **Returns:**: the revision

---


### buildEvaluator

```
public void buildEvaluator(Instances data)
                    throws java.lang.Exception
```

:   Initializes a filtered attribute evaluator.

    :   **Specified by:**: `buildEvaluator` in class `ASEvaluation`
    :   **Parameters:**: `data` - set of instances serving as training data **Throws:**: `java.lang.Exception` - if the evaluator has not been generated successfully

---


### evaluateSubset

```
public double evaluateSubset(java.util.BitSet subset)
                      throws java.lang.Exception
```

:   evaluates a subset of attributes

    :   **Specified by:**: `evaluateSubset` in interface `SubsetEvaluator`
    :   **Parameters:**: `subset` - a bitset representing the attribute subset to be evaluated **Returns:**: the "merit" of the subset **Throws:**: `java.lang.Exception` - if the subset could not be evaluated

---


### toString

```
public java.lang.String toString()
```

:   Describe the attribute evaluator

    :   **Overrides:**: `toString` in class `java.lang.Object`
    :   **Returns:**: a description of the attribute evaluator as a string

---


### main

```
public static void main(java.lang.String[] args)
```

:   Main method for testing this class.

    :   **Parameters:**: `args` - the options


---


|  |  |  |
| --- | --- | --- |
| **Overview** \| **Package** \| **Class** \| **Tree** \| **Deprecated** \| **Index** \| **Help** \| **Weka's home** | |  |
| **PREV CLASS**   **NEXT CLASS** | **FRAMES**    **NO FRAMES**     **All Classes** |  |
| SUMMARY: NESTED \| FIELD \| CONSTR \| METHOD | DETAIL: FIELD \| CONSTR \| METHOD |  |


---
